# Esculentoside A Inhibits Proliferation, Colony Formation, Migration, and Invasion of Human Colorectal Cancer Cells

**DOI:** 10.1155/2023/7530725

**Published:** 2023-02-10

**Authors:** Maha Abdullah Momenah, Layla Awad Almutairi, Haifa Ali Alqhtani, Fatimah A. Al-Saeed, Khalid M. Al Syaad, Sadeq K. Alhag, Mohammed A. Al-qahtani, Zaki Hussain Hakami, Jewel Mallick, Ahmed Ezzat Ahmed

**Affiliations:** ^1^Department of Biology, College of Science, Princess Nourah bint Abdulrahman University, P.O. Box 84428, Riyadh 11671, Saudi Arabia; ^2^Department of Biology, College of Science, King Khalid University, Abha 61413, Saudi Arabia; ^3^Biology Department, Faculty of Science, King Khalid University, P.O. Box 9004, Abha 61413, Saudi Arabia; ^4^Director of the Research Center, Faculty of Science, King Khalid University, P.O. Box 9004, Abha 61413, Saudi Arabia; ^5^Biology Department, College of Science and Arts, King Khalid University, Muhayl Asser, Saudi Arabia; ^6^Department of Medical Laboratory Technology, Faculty of Applied Medical Sciences, Jazan University, Jazan, Saudi Arabia; ^7^Department of Pharmacy, BGC Trust University Bangladesh, Chittagong 4381, Bangladesh; ^8^Department of Biology, College of Science, King Khalid University, P.O. Box 9004, Abha 61413, Saudi Arabia; ^9^Prince Sultan Bin-Abdul-Aziz Center for Environment and Tourism Studies and Researches, King Khalid University, P.O. Box 960, Abha 61421, Saudi Arabia; ^10^Department of Theriogenology, Faculty of Veterinary Medicine, South Valley University, Qena 83523, Egypt

## Abstract

Esculentosides include a group of plant-derived compounds with tremendous pharmacological potential. The antiproliferative effects of esculentoside A against different colorectal cancer cells were evaluated. We found that the proliferation of all the colorectal cancer cells was halted by esculentoside A. The IC_50_ of esculentoside A ranged from 16 to 24 *μ*M against different colorectal cancer cells. Investigation of the underlying molecular mechanism revealed that esculentoside A caused an increase in the colorectal cancer cells at the G1 phase of the cell cycle, indicative of G0/G1 cell cycle arrest. The percentage of G1 cells increased from 22.68% in control to 54.23% at 16 *μ*M esculentoside A. We also found that the colony formation of HT-29 cells was inhibited by 59% at 24 *μ*M esculentoside A. Finally, effects of esculentoside A on the motility of HT-29 colorectal cancer cells were investigated, and it was found that esculentoside A caused a significant decline in HT-29 colorectal cancer cell migration and invasion. The migration and invasion of esculentoside A-treated HT-29 cells were 45% and 51% higher, respectively, than those of untreated cells. Summing up, these results suggest that esculentoside A exhibits antiproliferative effects against human colorectal cancer cells.

## 1. Introduction

Esculentosides constitute a large and diverse group of oleanene-type saponins with a wide array of pharmacological properties [1]. They are generally isolated from plant species belonging to the family *Phytolaccaceae*. Plant species such as *Phytolacca esculenta* and *Phytolacca americana* are important sources of esculentosides [2, 3]. They have been shown to have diverse bioactivities, which include antimicrobial, anti-inflammatory, and anticancer properties, to name a few [1–3]. Esculentoside A is an important saponin that has been shown to suppress the growth of several cancer types. Liu et al. showed that esculentoside A halts the growth of human breast cancer cells by inducing apoptotic cell death [4]. They showed that esculentoside A exhibits this property *b* by blocking the IL‐6/STAT3 signaling [4]. However, esculentoside A has been evaluated for its anticancer properties against human colorectal cancer cells (CCC). Annually, colorectal cancer has been reported to cause around 0.8 million deaths and is currently one of the most predominant cancer types in both men and women [5]. It has been predicted that the burden of colon cancer will keep on increasing if there is no development in early detection and if no effective interventions for advanced staged colorectal cancer are made [6]. At present, routine colonoscopy and subsequent surgical resection of the tumors are the primary innervational options available for colorectal cancer patients. The chemotherapeutic drugs have many side effects, and the disease often relapses, making it very difficult to manage [7]. Accordingly, research efforts are being put into detecting the disease at an early stage and developing potential chemotherapeutic agents for the management of colorectal cancer. Herein, we evaluated the growth inhibitory effects of esculentoside A against human colorectal cancer cells. It was revealed that esculentoside A halted the growth of cancer cells and inhibited their colony formation and metastasis. We believe that this investigation will play an important role in establishing esculentoside A as a lead molecule for colorectal cancer.

## 2. Materials and Methods

### 2.1. Cell Lines and Culturing

CCC lines (HCT-116, HT-29, and SW620) were bought from ATCC and cultured in DMEM with 10% fetal bovine serum, 1% streptomycin/penicillin at 37°C, with 5% CO_2_.

### 2.2. Cell Viability Assay

Cell counting kit-8 assay was used to evaluate the viability of colorectal cancer cells (HCT-116, HT-29, and SW620). At the density of cells (2 × 10^4^ cells per well), the cells were put in 96-well plates and administered with varied dosages of esculentoside A. Subsequently, 10 *μ*L CCK-8 solution was supplemented to the cells, which were then incubated for 1 h at 37°C. After this, the optical density was determined at 450 nm using an ELISA plate reader.

### 2.3. Colony Formation Assay

In the case of the colony-forming assay, the culturing of HT-29 cells was done.

10 mL of culturing medium with 5000 cells per dish. Then, culturing of the cells was done for 2 weeks. After the colonies became visible, they fixed them using crystal violet for 15 min and photographed them.

### 2.4. Cell Cycle Analysis

HT-29 cells were subjected to fixation with 70% ethanol at 4°C for 12 h. Afterwards, a 100 *μ*L suspension was treated with 50 *μ*g propidium iodide (PI) at 4°C for 35 min. Lastly, cell cycle phase distribution was estimated by using a flow cytometer. 15,000 cells/sample were taken and analysed by BD FACSuite software version 1.0.

### 2.5. Transwell Assay

Migration and invasion of cells were estimated using Transwell chambers (BD Biosciences) with either Matrigel coating or without it for cell invasion and migration, respectively. HT-29 cells were put into the upper chambers. Nonetheless, lower chambers were filled with 10% FBS-containing medium. Cells were then incubated for 24 h and passed via membranes that were stained with 0.1% crystal violet (Sangon Biotech).

### 2.6. Statistical Analysis

Experimental procedures were done in triplicate. Data are presented as mean ± standard deviation (SD). For statistical analysis, Student's *t*-test with *P* < 0.05 was used.

## 3. Results

### 3.1. Esculentoside A Inhibits Proliferation of Colorectal Cancer Cells

**Figure 1:**
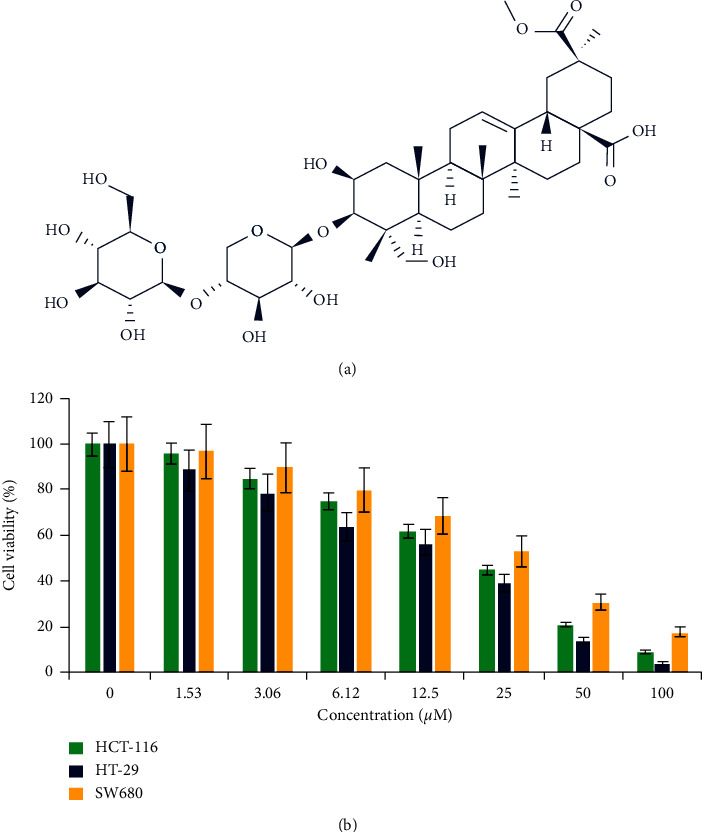
Esculentoside A exerts antiproliferative effects on colorectal cancer cells. (a) Esculentoside A structure. (b) Effect of esculentoside A on the viability of colorectal cancer cells. Experiments were done in triplicate.

Effects of esculentoside A (Figure 1(a)) on the proliferation of HT-29, HCT-116, and SW620 cell lines by the CCK-8 assay showed that esculentoside A triggered growth inhibitory effects on all three colorectal cancer cell lines (Figure 1(b)). These growth inhibitory effects of esculentoside A were found to be dose-dependent. The IC_50_ of esculentoside A ranged between 16 and 24 *μ*M. The lowest IC_50_ of 16 *μ*M was observed against the HT-29 cell line. As a result, this cell line was used for next experiments.

### 3.2. Esculentoside A Induces Cell Cycle Arrest of Colorectal Cancer Cells

**Figure 2:**
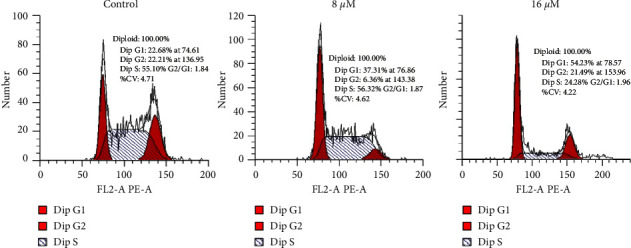
Esculentoside A induces cell arrest of colorectal cancer cells. Flow cytometry showing HT-29 cells distribution at various phases of cell cycle. The experiments were carried out in triplicate.

Effects of Esculentoside A were assessed on HT-29 cells' distribution in phases of the cell cycle. We found that the esculentoside A of HT-29 cells triggered their accumulation at the G1 phase of the cell cycle. The percentage of the G1 phase cells was enhanced from 22.68% to 54.23% at 16 *μ*M dosage of esculentoside A (Figure 2). These findings are indicative of G0/G1 cell cycle arrest.

### 3.3. Esculentoside A Inhibits Colony Formation of Colorectal Cancer Cells

**Figure 3:**
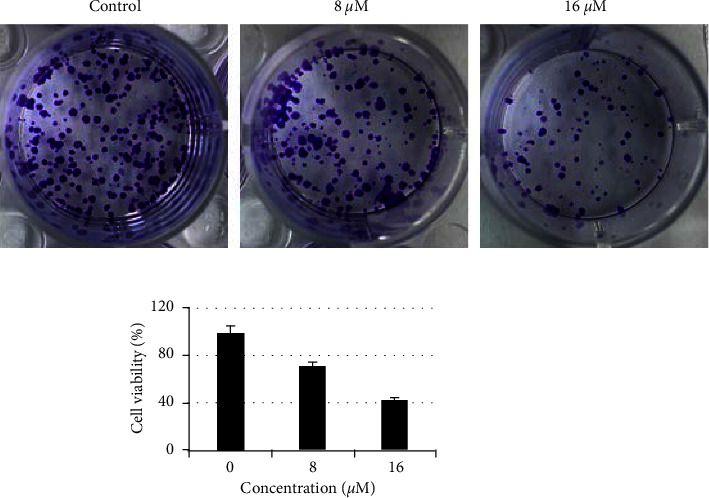
Esculentoside A inhibits formation of colorectal cancer cell colonies. Colony formation assay showing the effect of various concentrations of esculentoside A on the colony formation potential of HT-29 cells. Experiments were repeated thrice.

The effects of esculentoside on the colony forming property of HT-29 cells were also examined. HT-29 cells were administrated with different dosages of esculentoside A and subsequently incubated at 37°C for 14 days. We found that esculentoside A diminished the colony forming property of HT-29 cells. At 16 *μ*M esculentoside A concentration, the colony formation was inhibited by 59% compared to the untreated HT-29 cells (Figure 3).

### 3.4. Esculentoside A Inhibits Migration and Invasion of Colorectal Cancer Cells

**Figure 4:**
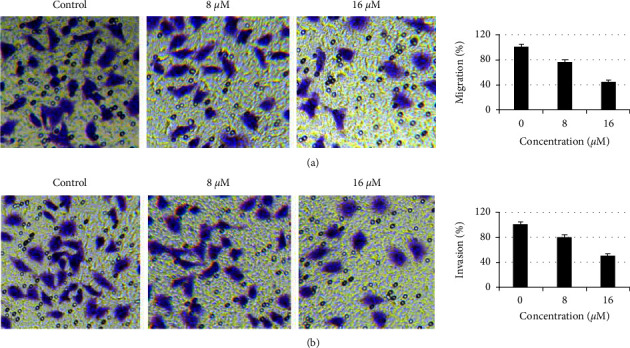
Esculentoside A inhibits colorectal cancer cell migration and invasion. The transwell assay showing the effect of different doses of esculentoside A on (a) migration and (b) invasion of HT-29 cells. Experiments were performed in triplicate.

Effects of esculentoside A on HT-29 cells migration and invasion were evaluated by the transwell assay. The results showed that the migration and invasion of esculentoside A-treated HT-29 cells were diminished in a dose-dependent manner. The migration and invasion of esculentoside A-treated HT-29 cells were 45% and 51% higher, respectively, than those of untreated cells (Figures 4(a) and 4(b)).

## 4. Discussion

Colorectal cancer is a devastating disease, causing a huge number of human mortalities across the world every year [8]. A lot of research efforts are put in from different corners of the world to decrease the incidence of colorectal cancer. However, due to the lack of reliable procedures for early detection and efficient chemotherapeutic agents without adverse effects, the management of colorectal cancer has become a mammoth task [9]. Researchers are looking for anticancer drugs from plant sources, as many previously used anticancer drugs have also come from plants [10]. Many plants are edible by nature, and it is believed that anticancer agents from such plants may prove to be nontoxic and safe [11–13]. In this study, a plant-derived saponin, esculentoside A, was evaluated for its antiproliferative effect against human colorectal cancer cells. Esculentoside A suppressed the growth of all the colorectal cancer cells. Liu et al. showed that esculentoside A targets the IL‐6/STAT3 cascade to halt the growth of breast cancer cells [4]. Many related compounds, such as esculentoside H, have been shown to block the NF-kB signaling cascade and suppress CCC proliferation. Cell cycle analysis showed that esculentoside A arrests the cells at the G0/G1 phase [14]. Several saponins have previously been shown to induce cell cycle arrest; for example, Pennogenyl saponins triggered cell cycle arrest in hepatocellular carcinoma cells [15]. Previously, migration and invasion of colon cancer cells were inhibited by esculentoside H [14]. In this study, the effects of esculentoside A on the migration and invasion of colorectal cancer cells were evaluated. Interestingly, the migration and invasion of colorectal cancer cells were blocked by esculentoside A treatment, indicative of its antimetastatic potential [16–18].

## 5. Conclusion

Esculentosides are plant-derived compounds that have tremendous pharmacological applications. The present investigation showed that esculentoside was able to suppress the proliferation and colony formation of colorectal cancer cells via G0/G1 cell cycle arrest. Furthermore, esculentoside A also reduced the movement and invasion of human colorectal cancer cells. Therefore, esculentoside A may prove to be a potential lead molecule for colorectal cancer chemotherapy. However, in vivo studies are required for further confirmation.

## Data Availability

All data used to support the findings of this study are included within the article.

## References

[1] Bailly C., Vergoten G. (2020). Esculentosides: insights into the potential health benefits, mechanisms of action and molecular targets. *Phytomedicine*.

[2] Yu H., Gong L., Wang X. (2016). Rabbit conjunctivae edema and release of NO, TNF-*α*, and IL-1*β* from macrophages induced by fractions and esculentosides isolated from Phytolacca americana. *Pharmaceutical Biology*.

[3] Gong W., Jiang Z., Sun P. (2011). Synthesis of novel derivatives of esculentoside A and its aglycone phytolaccagenin, and evaluation of their haemolytic activity and inhibition of lipopolysaccharide induced nitric oxide production. *Chemistry and Biodiversity*.

[4] Liu C., Dong L., Sun Z. (2018). Esculentoside A suppresses breast cancer stem cell growth through stemness attenuation and apoptosis induction by blocking IL 6/STAT3 signaling pathway. *Phytotherapy Research*.

[5] Siegel R. L., Miller K. D., Goding Sauer A. (2020). Colorectal cancer statistics, 2020. *CA: A Cancer Journal for Clinicians*.

[6] Hafez N., Gettinger S. (2020). Oligometastatic disease and local therapies: a medical oncology perspective. *The Cancer Journal*.

[7] Douillard J. Y., Siena S., Cassidy J. (2014). Final results from PRIME: randomized phase III study of panitumumab with FOLFOX4 for first-line treatment of metastatic colorectal cancer. *Annals of Oncology*.

[8] Center M. M., Jemal A., Smith R. A., Ward E. (2009). Worldwide variations in colorectal cancer. *CA: A Cancer Journal for Clinicians*.

[9] Fearon E. R. (2011). Molecular genetics of colorectal cancer. *Annual Review of Pathology: Mechanisms of Disease*.

[10] Pezzuto J. M. (1997). Plant-derived anticancer agents. *Biochemical Pharmacology*.

[11] Iqbal J., Abbasi B. A., Mahmood T. (2017). Plant-derived anticancer agents: a green anticancer approach. *Asian Pacific Journal of Tropical Biomedicine*.

[12] Cragg G. M., Newman D. J. (2005). Plants as a source of anti-cancer agents. *Journal of Ethnopharmacology*.

[13] Shah U., Shah R., Acharya S., Acharya N. (2014). Novel anticancer agents from plant sources. *Chinese Journal of Natural Medicines*.

[14] Ha S. H., Kwon K. M., Park J. Y. (2019). Esculentoside H inhibits colon cancer cell migration and growth through suppression of MMP 9 gene expression via NF kB signaling pathway. *Journal of Cellular Biochemistry*.

[15] Long F. Y., Chen Y. S., Zhang L. (2015). Pennogenyl saponins induce cell cycle arrest and apoptosis in human hepatocellular carcinoma HepG2 cells. *Journal of Ethnopharmacology*.

[16] Mitra S., Lami M. S., Ghosh A. (2022). Hormonal therapy for gynecological cancers: how far has science progressed toward clinical applications?. *Cancers*.

[17] Rahman M. M., Islam F., Afsana Mim S. (2022). Multifunctional therapeutic approach of nanomedicines against inflammation in cancer and aging. *Journal of Nanomaterials*.

[18] Islam M. R., Islam F., Nafady M. H. (2022). Natural small molecules in breast cancer treatment: understandings from a therapeutic viewpoint. *Molecules*.

